# Integrated Analysis of *C16orf54* as a Potential Prognostic, Diagnostic, and Immune Marker across Pan-Cancer

**DOI:** 10.1155/2022/9365046

**Published:** 2022-09-09

**Authors:** Xinna Du, Wei Xia, Weiping Fan, Xuan Shen, Hongyan Wu, Hu Zhang

**Affiliations:** ^1^Department of Pathology, Jiangsu Vocational College of Medicine, Yancheng, China; ^2^Institute of Pharmaceutical Biotechnology, Jiangsu Vocational College of Medicine, Yancheng, China; ^3^Department of Physiology and Biochemistry, Jiangsu Vocational College of Medicine, Yancheng, China

## Abstract

*Chromosome 16 open reading frame 54* (*C16orf54*) is a protein coding gene, showing a biased expression in the bone marrow, lymph node, and 11 other tissues. Reports on the function of *C16orf54* in the onset and development of tumours remain scarce. Clinical information and tumour expression profile data from The Cancer Genome Atlas (TCGA), Cancer Cell Line Encyclopedia (CCLE), and Genotype-Tissue Expression (GTEx) were utilized to determine the relationship between *C16orf54* expression and prognosis, diagnosis, immune microenvironment, heterogeneity, and stemness across pan-cancer. The findings ascertained that *C16orf54* was expressed at a low level in most cancers. Furthermore, *C16orf54* could distinguish between cancer and normal tissues with high accuracy in most cancers, and the prognostic significance of low *C16orf54* mRNA levels differs across cancers. *C16orf54* expression was positively linked to the stromal, immune, and ESTIMATE scores. On the other hand, *C16orf54* was reported to be negatively correlated with tumour purity in most cancers. Further, *C16orf54* expression was positively correlated with immune cell infiltration and the expression of immune regulatory genes, including chemokines, receptors, major histocompatibility complexes, immune inhibitory, and immune stimulatory genes, in most cancers. Additionally, *C16orf54* expression was significantly associated with tumour heterogeneity indicators, such as tumour mutation burden (TMB) and microsatellite instability (MSI), and was significantly correlated with DNAss and RNAss tumour stemness indicators. Moreover, Kyoto Encyclopaedia of Genes and Genomes (KEGG) analysis, as well as Gene Set Enrichment analysis (GSEA), revealed that *C16orf54* expression was closely linked to the signalling pathways of immune cells and factors. The integrated analysis of *C16orf54* indicates it as a potential prognostic, diagnostic, and immune marker, which could be adopted as a novel target for adjuvant immunotherapy across pan-cancer.

## 1. Introduction

The latest global cancer burden data issued by the International Agency for Research on Cancer (IARC) reported 19.29 million new malignancy individuals and nearly 10 million cancer-related mortalities globally in 2022 [1]. Reducing the cancer mortality rate remains a major hurdle as currently there is no absolute cure for cancer.

Recently, tumour immunotherapy, especially the advent of immune checkpoint inhibitors, has become another prominent and effective tumour treatment measure. Different from traditional chemotherapy and targeted therapy, immune checkpoint inhibition therapy does not directly act on tumour cells, but on the immune cells. It can recognize and kill tumour cells by restoring the previously “suppressed” immune system. The emergence of immunotherapy has greatly improved current cancer treatments. However, the interaction between tumour cells and the immune system is a continuous, dynamic, and evolving process. Furthermore, tumour malignancy largely depends on the development of the immune escape function of tumour cells [2], which hinders the efficacy of cancer immunotherapy. The main mechanism of tumour cell immune escape is the reduction of cytokine (CXCL9, CXCL10, and CCL3) secretion and killer immune cells, that is, T cells and natural killer (NK) cell infiltration levels. The increased secretion of IL-6, IL-33, and CXCL7 and the high expression of PD-L1 immune checkpoint ligand inhibit the function of T cells and reduce their infiltration. Various tumours have the potential to inhibit the effective recognition and killing of tumour cells by the immune system via various pathways, thereby producing immune tolerance and promoting tumour occurrence and development. The identification of biomarkers that can accurately predict the treatment response, aid in formulating individualised treatment regimens, and reduce the injury and burden caused by overtreatment and improper treatment is not only an urgent clinical need but also the biggest challenge faced by tumour immunotherapy [3–5].

Tumour multiomics databases, including The Cancer Genome Atlas (TCGA), offer endless possibilities for identifying novel prognostic, diagnostic, and immunotherapy relevant markers. *Chromosome 16 open reading frame 54* (*C16orf54*) is a protein coding gene which located at 16p11.2 and is expressed in 11 normal tissues, such as the bone marrow and lymph node. Moreover, the encoded protein is mainly located in the cell membrane. To the best of our knowledge, the role of *C16orf54* in tumourigenesis, development, and immune remains unknown. In this study, multiple bioinformatics tools are used to integrate multiple multiomics high-throughput data, such as TCGA data, to analyse the differential expression of *C16orf54* and its prognostic and diagnostic roles in pan-cancer. Furthermore, the link between *C16orf54* expression and tumour immune microenvironment (TIME), tumour heterogeneity, and stemness are analysed. Additionally, the possible molecular mechanism of *C16orf54* across pan-cancer is elucidated using Kyoto Encyclopaedia of Genes and Genomes (KEGG) analysis and Gene Set Enrichment analysis (GSEA).

## 2. Methods

### 2.1. Data Processing and Pan-Cancer Analysis of C16orf54 Expression

Normalised expression profile data and clinical data from TCGA and Genotype-Tissue Expression (GTEx) were retrieved from the website known as UCSC XENA (https://xenabrowser.net/datapages/) [6]. The Cancer Cell Line Encyclopedia (CCLE) data were downloaded from the DepMap portal (https://depmap.org/portal/download/). Besides, *C16orf54* expression profile data were log2 transformed for comparison between groups. The Simple Nucleotide Variation dataset of level 4 of every TCGA sample was processed using the MuTect2 software and such data were downloaded from GDC (https://portal.gdc.cancer.gov/). The tmb function of the R software package “maftools” was used to calculate each tumour mutation burden (TMB). Based on the previous study [7], we obtained the microsatellite instability (MSI) score and the purity data for every tumour. Similarly, tumour stemness scores of DNAss and RNAss for each tumour were obtained from a previous study [8].

The R software (version 3.6.4) was used to compute *C16orf54* expression across pan-cancer, and “ggplot2” was used for visualisation. The immunohistochemical results of C16orf54 protein were queried and downloaded from the Human Protein Atlas (HPA) website (https://www.proteinatlas.org/). *p* < 0.05 indicated statistical significance for the difference between tumour and normal tissues.

### 2.2. Prognostic and Diagnostic Significance Analysis

R software package “survival” for Cox regression analysis was utilized to study the relationship between *C16orf54* expression and the prognosis of tumour patients, such as overall survival (OS), disease-specific survival (DSS), disease-free interval (DFI), and progression-free interval (PFI). Additionally, the prognostic differences between the two groups were analysed using “survminer” and “survival” R packages.

The potential value of *C16orf54* expression in each tumour diagnosis was analysed using the R software package “pROC” and visualised using the “ggplot2” package.

### 2.3. Tumour Microenvironment (TME) Analysis

The stromal, immune, and ESTIMATE scores of each patient were calculated using the R software package “ESTIMATE.” The purity data of each tumour were obtained from a previous study [9], and the corr.test function of the R software package “psych” was used to calculate the Pearson's correlation coefficient of *C16orf54* expression in each tumour along with the immune infiltration score and tumour purity.

### 2.4. Analysis of Immune Cell Infiltration

The link between *C16orf54* expression and each immune cell infiltration (*p* < 0.05) was analysed using the TIMER2.0 (http://timer.cistrome.org/) platform [10].

### 2.5. Analysis of Tumour Immunoregulatory Genes

Pearson's correlation coefficient of *C16orf54* and immunoregulatory gene expression in each tumour [11] was analysed using the corr.test function of the R software package “psych.”

### 2.6. Analysis of Tumour Heterogeneity and Stemness

Pearson's correlation coefficient between *C16orf54* expression and TMB, MSI, DNAss, and RNAss in each tumour was computed using the corr.test function found in the R software package “psych.”

### 2.7. C16orf54 Functional Enrichment Analysis

The biological significance of *C16orf54* expression in tumours was elucidated using the KEGG signal pathway analysis [12] and GSEA [13].

The R software package “stat” was utilized to compute the link between protein-coding genes and *C16orf54* expression in each tumour, and the top 300 genes that positively linked to *C16orf54* expression were obtained. The “clusterProfiler” and “org.Hs.eg.db” packages were used for KEGG enrichment analysis, using the “ggplot2” package for visualisation.

GSEA was performed using the “clusterProfiler” package, wherein the samples were categorized into two groups based on the *C16orf54* expression levels, and the h.all.v7.4.symbols.gmt subset was retrieved from the Molecular Signatures Database to ascertain the correlation between pathways and molecular mechanisms [14], *p*.adjust < 0.05, and false discovery rate (FDR) < 0.25 were statistically significant.

### 2.8. Statistical Analyses

The difference in *C16orf54* expression between normal tissues and different tumour cell lines was analysed with the aid of Kruskal–Wallis test. Pan-cancer analysis was performed utilizing unpaired Wilcoxon Rank Sum as well as Signed Rank Tests for significant difference analysis. Cox regression and log-rank tests were conducted to analyse the differences in survival time distribution in different groups. The link between *C16orf54* expression and immune indexes was analysed by the Pearson's test (no significant (ns)); *p* ≥ 0.05; ^∗^*p* < 0.05; ^∗∗^*p* < 0.01; ^∗∗∗^*p* < 0.001. The findings demonstrated that the area under the curve (AUC) value in the *receiver operating characteristic* (ROC) curve was closer to 1, indicating better diagnostic effects. AUC has low, certain, and high accuracy when it falls within 0.5–0.7, 0.7–0.9, and above 0.9, respectively.

## 3. Results

### 3.1. Differential Expression of C16orf54 across Pan-Cancer

**Figure 1:**
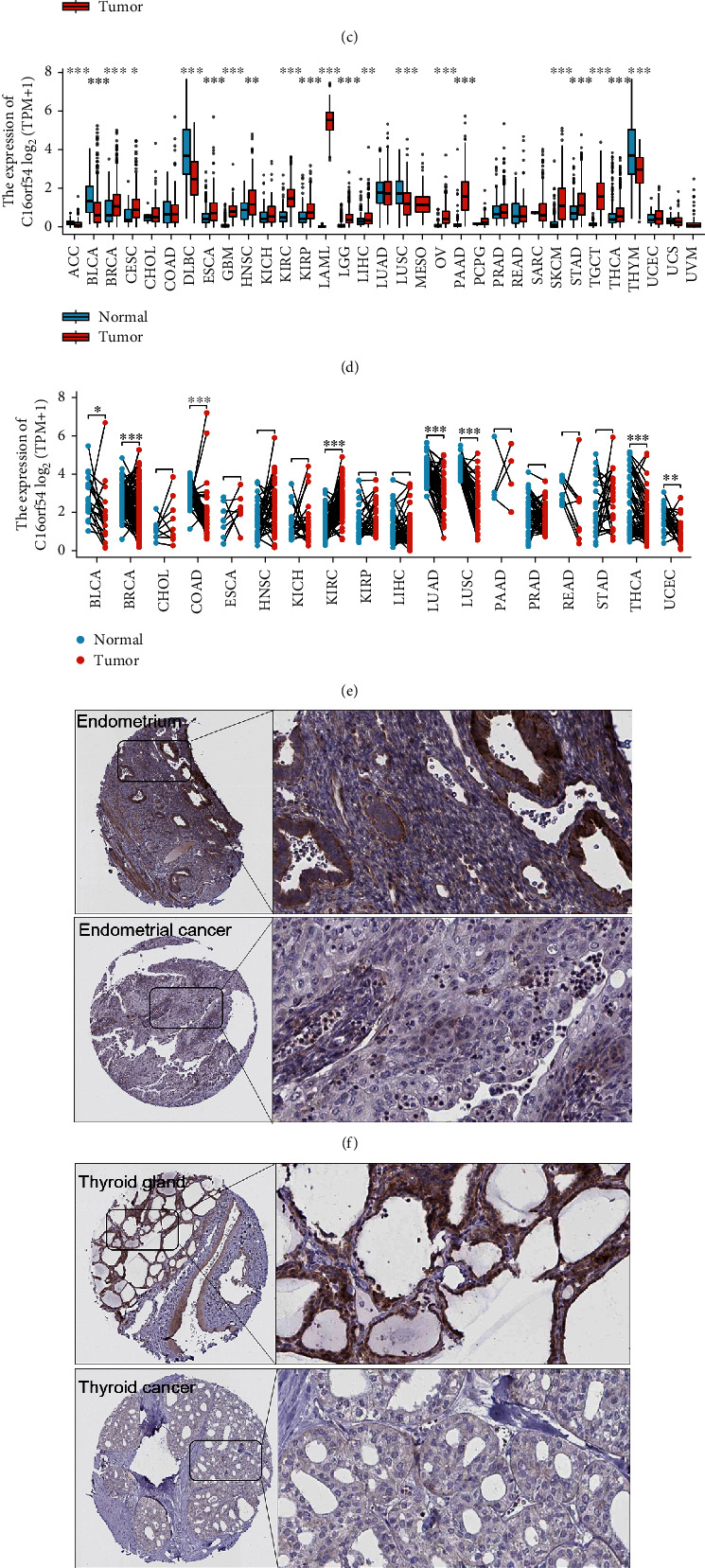
Differential expression of *C16orf54*. (a) *C16orf54* expression in normal tissues. (b) *C16orf54* expression in tumour cell lines. (c) Expression analysis of *C16orf54* by TCGA data, and (d) integrated data of TCGA and GTEx. (e) Expression analysis of *C16orf54* in paired samples. Immunohistochemical results of C16orf54 in (f) endometrium, endometrial cancer, (g) thyroid gland, thyroid cancer, (h) liver, liver cancer, and (i) urinary bladder and urothelial cancer. ^∗^*p* < 0.05; ^∗∗^*p* < 0.01; ^∗∗∗^*p* < 0.001.

*C16orf54* expression was analysed across pan-cancer and normal tissues. Different tissues from GTEx showed significantly different *C16orf54* expression (Figure 1(a), *p* < 0.001), with high expression in the blood, spleen, small intestine, and other tissues. *C16orf54* expression in different cell line from CCLE was also significantly different (*p* < 0.001), with higher expression in haematopoietic and lymphatic cells (Figure 1(b), *p* < 0.001). TCGA data analysis revealed that *C16orf54* expression was substantially upregulated in glioblastoma multiforme (GBM), head and neck squamous cell carcinoma (HNSC), and kidney renal clear cell carcinoma (KIRC), whereas it was downregulated in bladder urothelial carcinoma (BLCA), breast invasive carcinoma (BRCA), colon adenocarcinoma (COAD), lung squamous cell carcinoma (LUSC), rectum adenocarcinoma (READ), lung adenocarcinoma (LUAD), uterine corpus endometrial carcinoma (UCEC), and thyroid carcinoma (THCA) compared to the corresponding samples from normal tissues (Figure 1(c)). TCGA integrated GTEx data showed that the *C16orf54* gene was significantly upregulated in BRCA, cervical squamous cell carcinoma and endocervical adenocarcinoma (CESC), GBM, KIRC, ovarian serous cystadenocarcinoma (OV), HNSC, kidney renal papillary cell carcinoma (KIRP), brain lower grade glioma (LGG), liver hepatocellular carcinoma (LIHC), acute myeloid leukemia (LAML), pancreatic adenocarcinoma (PAAD), stomach adenocarcinoma (STAD), testicular germ cell tumours (TGCT), skin cutaneous melanoma (SKCM), esophageal carcinoma (ESCA), and THCA but downregulated in BLCA, adrenocortical carcinoma (ACC), lymphoid neoplasm diffuse large B-cell lymphoma (DLBC), LUSC, and thymoma (THYM) compared to their respective samples from normal tissues (Figure 1(d)). The analysis of paired samples revealed that *C16orf54* was substantially upregulated in KIPC but significantly downregulated in BRCA, BLCA, COAD, LUSC, LUAD, UCEC, and THCA in (Figure 1(e)). Furthermore, immunohistochemical results showed that protein levels of C16orf54 in endometrial cancer, thyroid cancer, liver cancer, and urothelial cancer were decreased compared with those in normal tissues (Figures 1(f)–1(i)). Therefore, *C16orf54* expression in various tissues and tumours is heterogeneous.

### 3.2. The Prognostic and Diagnostic Value of *C16orf54*

To explore the clinical significance of *C16orf54* expression in tumours, the correlation between *C16orf54* expression and the prognosis of patients, including OS, DSS, DFI, and PFI, and diagnosis in each tumour was analysed.

**Figure 2:**
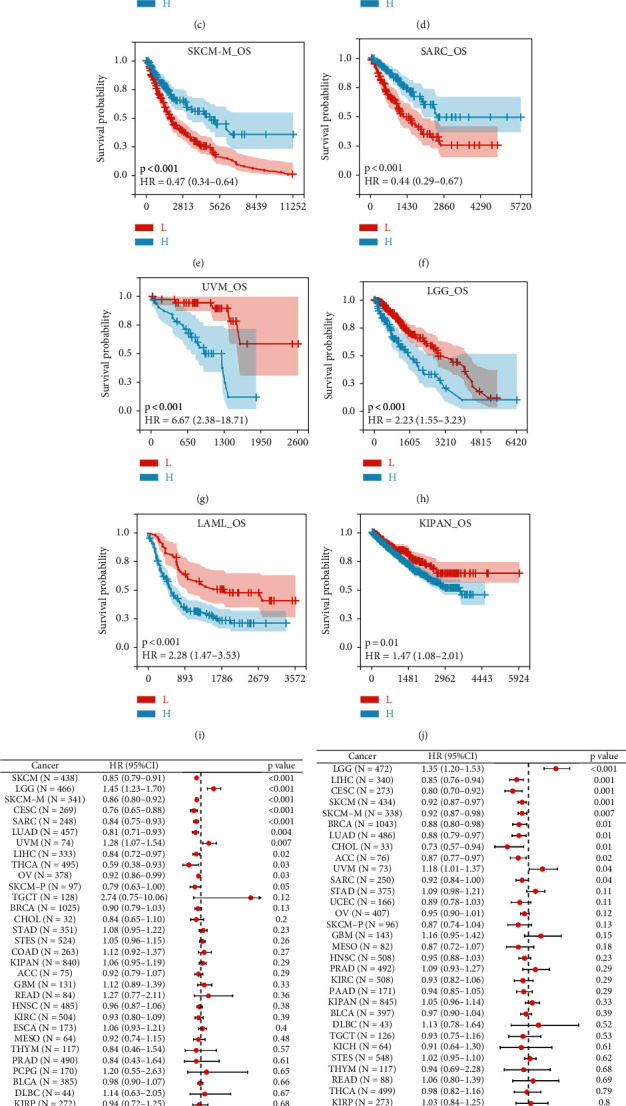
The prognostic value of *C16orf54* expression. (a) Forest plot of overall survival (OS) in 37 tumour types. (b–j) Kaplan–Meier analysis of the association between *C16orf54* expression and OS. (k) Forest plot of disease-specific survival (DSS) in 36 tumour types. (l) Forest plot of progression-free interval (PFI) in 36 tumour types. (m) Forest plot of disease-free interval (DFI) in 30 tumour types.

Cox proportional hazards regression model analysis confirmed that high *C16orf54* expression was related to poor OS in LGG, LAML, pan-kidney cohort (KIPAN), and uveal melanoma (UVM), whereas low expression was associated with a poor prognosis in SKCM, SKCM-M, sarcoma (SARC), CESC, LUAD, LIHC, and OV (Figure 2(a)). Kaplan–Meier analysis revealed that low *C16orf54* expression was related to poor OS in SKCM, LUAD, CESC, SKCM-M, and SARC (Figures 2(b)–2(f)), whereas high expression was associated with poor OS in UVM, LGG, LAML, and KIPAN (Figures 2(g)–2(j)). DSS analysis affirmed that the high expression of *C16orf54* was linked to poor prognosis in LGG and UVM, but low expression was associated with a poor prognosis in SKCM, SKCM-M, CESC, SARC, LUAD, LIHC, THCA, and OV (Figure 2(k)). Forest plots of PFI revealed that high *C16orf54* mRNA levels were associated with a poor prognosis in LGG and UVM while low levels were associated with a poor prognosis in LIHC, CESC, SKCM, SKCM-M, BRCA, LUAD, cholangiocarcinoma (CHOL), ACC, and SARC (Figure 2(l)). DFI analysis further revealed that low *C16orf54* expression was associated with a poor prognosis in LIHC and OV (Figure 2(m)). Additionally, Kaplan–Meier analyses demonstrate the correlation analysis between *C16orf54* expression and DSS, DFI, and PFI. DSS analysis revealed that low *C16orf54* expression was linked to a lower DSS in OV, SKCM, SKCM-M, SARC, CESC, and LUAD and a higher DSS in LIHC, UVM, and LGG (Supplementary Figure 1). DFI and PFI analyses showed that the low expression of *C16orf54* was associated with a lower PFI in CESC, SKCM, SKCM-M, SARC, LUAD, LIHC, BRCA, CESC, LGG, and UVM and a lower DFI in LIHC and OV (Supplementary Figure 2).

**Figure 3:**
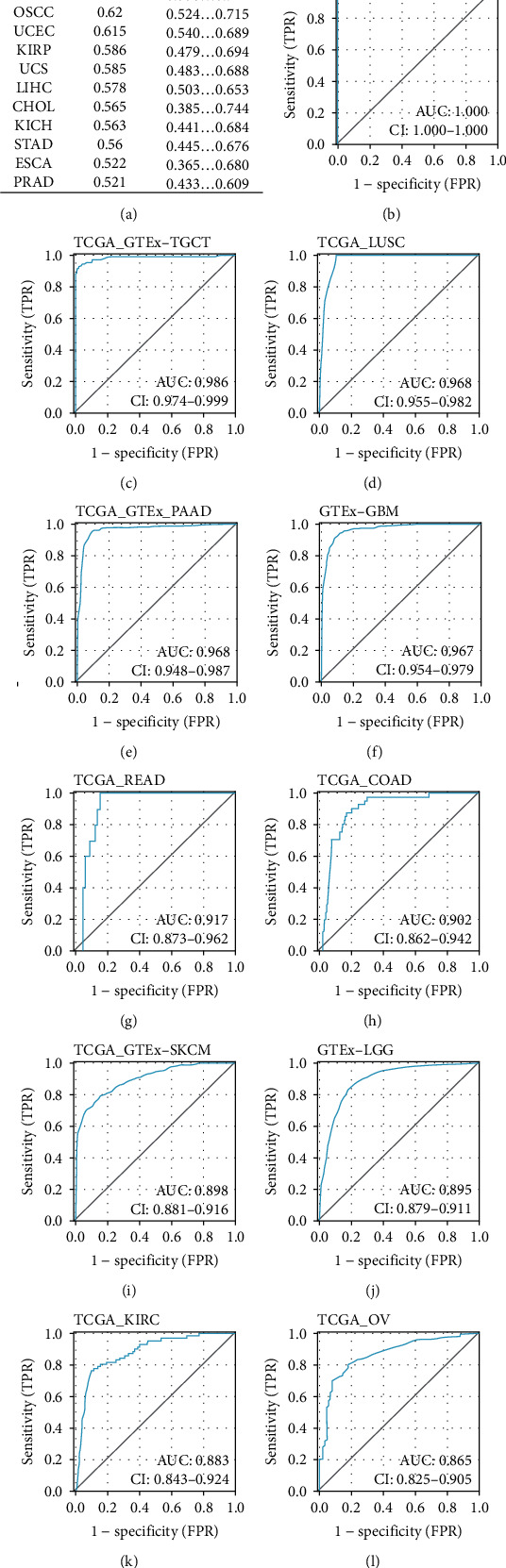
The ROC curve of *C16orf54*. (a) The ROC results of *C16orf54* across pan-cancer. (b–m) ROC curves of *C16orf54* in various tumours.

Additionally, the ROC curve analysis revealed that *C16orf54* had a high accuracy in anticipating tumour and normal outcomes in LAML (AUC = 1), testicular germ cell tumours (TCGT) (AUC = 0.986), LUSC (AUC = 0.968), PAAD (AUC = 0.968), GBM (AUC = 0.967), READ (AUC = 0.917), COAD (AUC = 0.902), had a certain accuracy in SKCM (AUC = 0.898), LGG (AUC = 0.895), KIRC (AUC = 0.883), OV (AUC = 0.865), LUAD (AUC = 0.84), BLCA (AUC = 0.759), and DLBC (AUC = 0.734), but lower accuracy in other tumours (Figure 3).

### 3.3. Correlation between the TME and *C16orf54* Expression

**Figure 4:**
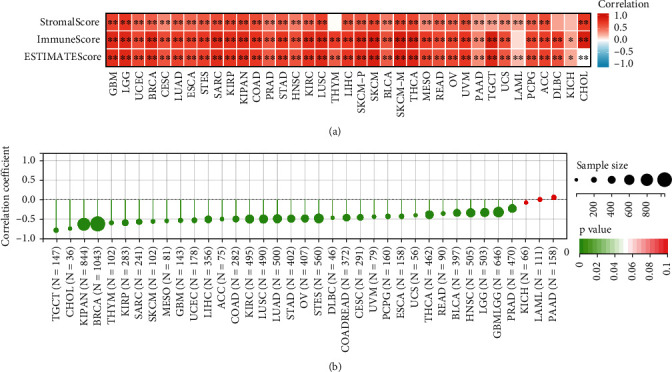
The correlation between *C16orf54* expression and TME. (a) Correlation between *C16orf54* and stromal, immune and ESTIMATE scores. (b) The correlation between *C16orf54* expression and tumour purity. ^∗^*p* < 0.05; ^∗∗^*p* < 0.01; ^∗∗∗^*p* < 0.001.

To explore the possible roles of *C16orf54* in TME, the link between *C16orf54* expression and TME index and tumour purity were analysed [15, 16]. *C16orf54* expression was found to be substantially positively related to immune indicators, including stromal, immune, and ESTIMATE scores (Figure 4(a)). Additionally, it was negatively linked to tumour purity in the majority of the tumours (Figure 4(b)).

### 3.4. Relationship between Immune Cell Infiltration and *C16orf54* Expression

**Figure 5:**
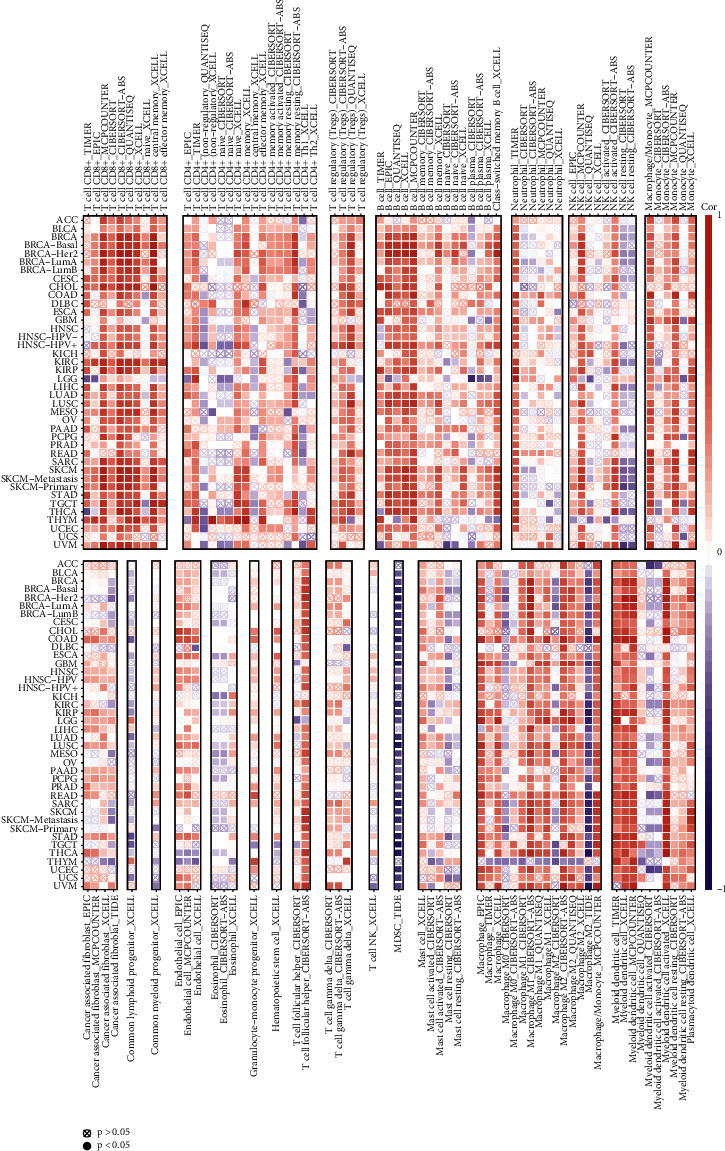
*C16orf54* expression and immune cell infiltration.

The relationship between *C16orf54* expression and immune cell infiltration levels in different tumour types was evaluated utilizing the TIMER website tool. This tool serves as a comprehensive resource for immune infiltrate-related systematic analysis across different cancer types [10]. Although the results of different algorithms may vary slightly, the overall trend remains the same. This study shows that *C16orf54* expression in the majority of tumour types was significantly positively linked to immune cell infiltration, including T cell CD4 +, T cell CD8 +, T cell regulatory (Tregs), B-cell, neutrophil, NK cell, monocyte, endothelial cell, macrophage, T cell follicular helper, and myeloid dendritic cell, and significantly negatively correlated with common lymphoid progenitor and eosinophil and myeloid-derived suppressor cells infiltration. Additionally, the correlation between *C16orf54* expression and the immune cell infiltration of cancer-associated fibroblast, common myeloid progenitor, granulocyte-monocyte progenitor, T cell gamma delta, hematopoietic stem cell, T cell NK and mast cell across pan-cancer was not significant (Figure 5).

### 3.5. Relationship between Immunoregulatory Genes and *C16orf54* Expression

**Figure 6:**
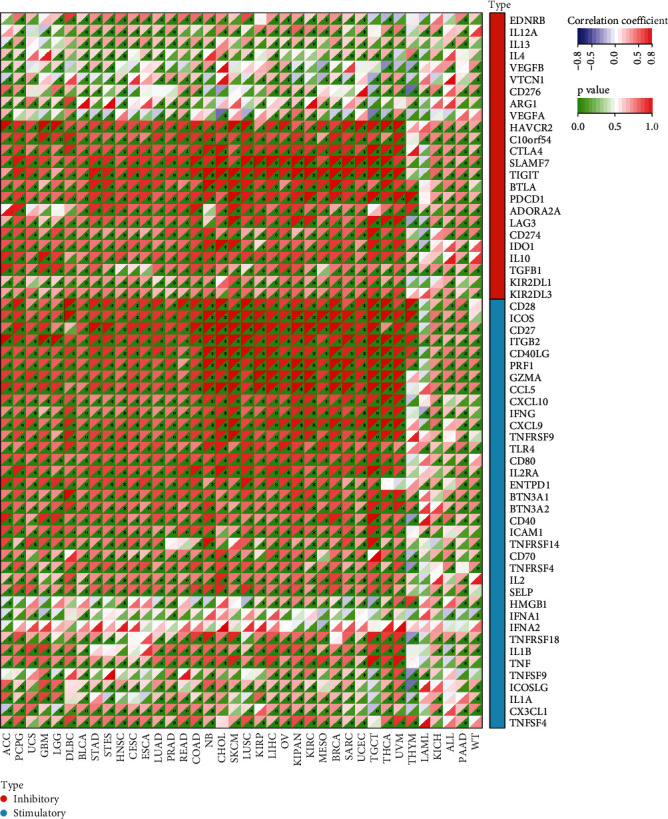
*C16orf54* expression and immune checkpoint genes. ^∗^*p* < 0.05.

**Figure 7:**
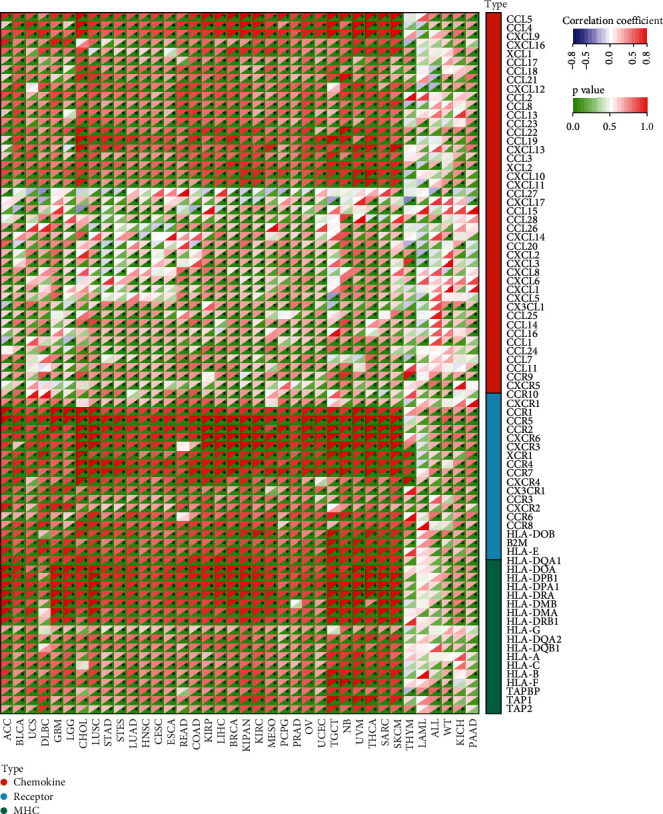
*C16orf54* expression and immunomodulatory genes. ^∗^*p* < 0.05.

To further investigate the role of *C16orf54* in TIME, the link between *C16orf54* expression and immunoregulatory genes in various tumour types was analysed. The Pearson's correlation coefficient between *C16orf54* expression and 60 immune checkpoint genes, including 24 inhibitory genes and 36 stimulatory genes (Figure 6), was calculated, which showed that almost all immune checkpoint genes, except IFNA1 and IFNA2, were significantly positively coexpressed with *C16orf54* in most tumours. Furthermore, the Pearson's correlation analysis of *C16orf54* and immunoregulatory genes (Figure 7), including 41 chemokine, 18 receptor, and 21 major histocompatibility complex (MHC), showed that *C16orf54* was significantly positively coexpressed with most immunomodulatory genes across pan-cancer.

### 3.6. Analysis of the Tumour Heterogeneity, Stemness, and *C16orf54* Expression

**Figure 8:**
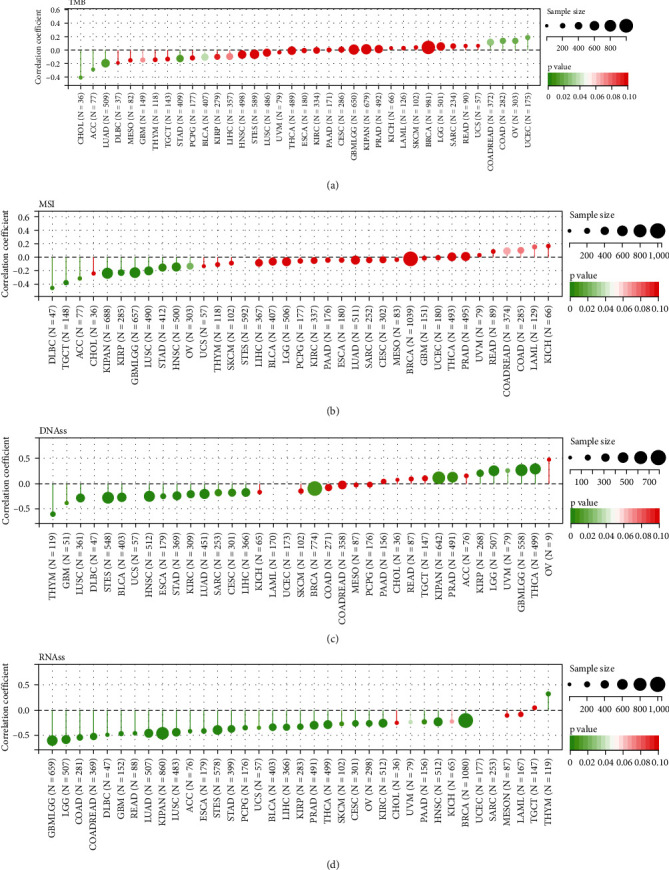
Analysis of tumour heterogeneity and stemness. (a) The relationship between *C16orf54* and mutational burden (TMB), (b) microsatellite instability (MSI), (c) DNAss, and (d) RNAss.

Tumour heterogeneity can modulate immunotherapy effects. Therefore, the Pearson's correlation coefficients between *C16orf54* and tumour heterogeneity indicators, TMB and MSI, were analysed. *C16orf54* expression was discovered to be significantly negatively related to TMB in CHOL, ACC, LUAD, STAD, and BLCA; significantly positively associated with TMB in CRC, COAD, OV, and UCEC (Figure 8(a)); and significantly negatively related to MSI in DLBC, TGCT, ACC, KIPAN, KIRP, GBM, LGG, LUSC, STAD, HNSC, and OV (Figure 8(b)).

Tumour stemness is not only associated with metastasis and heterogeneity, but also with immune checkpoint gene expression and cell infiltration of the immune system [8]. The analysis of *C16orf54* expression and tumour stemness indicators, DNAss and RNAss, suggested that *C16orf54* expression was substantially negatively correlated with DNAss in 15 tumour types (Figure 8(c)), including THYM, GBM, and LUSC; significantly positively correlated with DNAss in 7 tumour types, including LGG, KIRP, and THCA; significantly negatively correlated with RNAss in 30 tumour types (Figure 8(d)), including GBM, LGG, CESC, and LUAD; and significantly positively correlated with RNAss in THYM.

### 3.7. KEGG and GSEA Analysis

**Figure 9:**
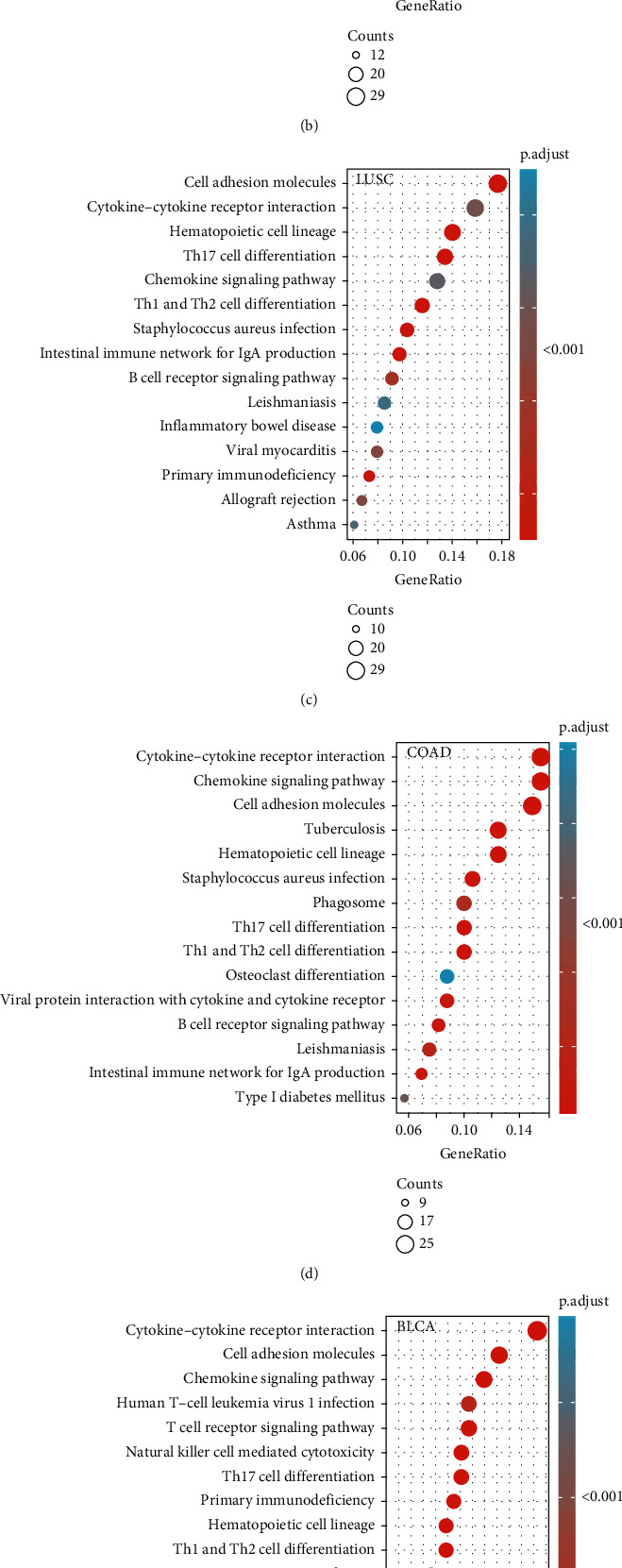
KEGG results of *C16orf54* in 6 selected tumours. The 15 typical signalling pathways in (a) KIRC, (b) LUAD, (c) LUCS, (d) COAD, (e) BLCA, and (f) READ.

**Figure 10:**
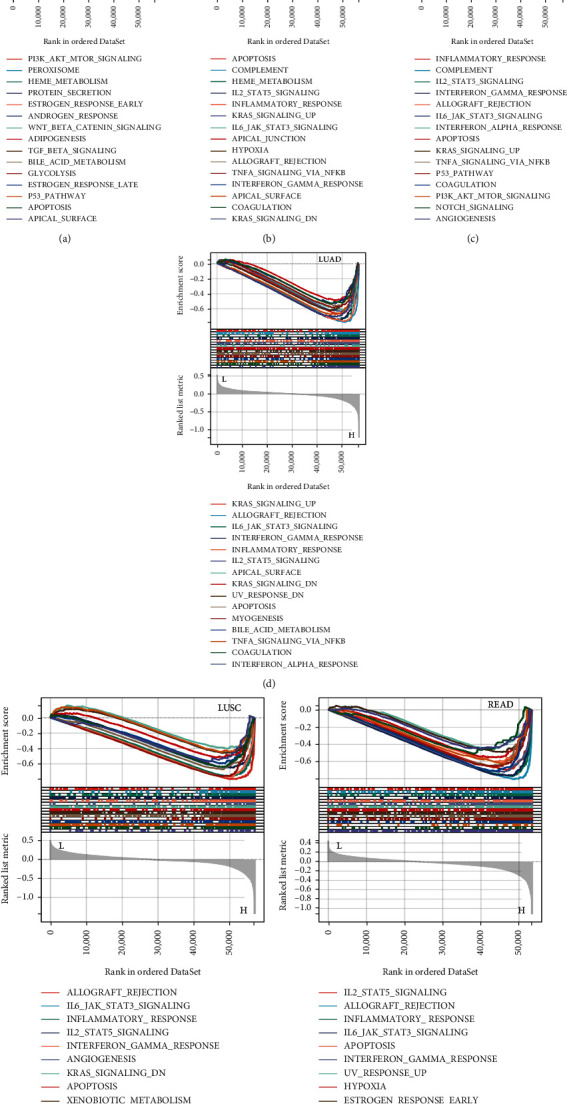
GSEA results of *C16orf54* in 6 selected tumours. The 15 typical signalling pathways in (a) BLCA, (b) COAD, (c) KIRC, (d) LUAD, (e) LUCS, and(f) READ.

To determine the biological significance of *C16orf54* in different tumour tissues, KEGG and GSEA analyses, were performed for 6 selected tumours. KEGG analysis revealed that *C16orf54* and its coexpressed top 300 genes mainly participated in the immune-related pathways of the cytokine-cytokine receptor interaction, Th1, Th2, and Th17 cell differentiation, T cell receptor signalling pathway, and hematopoietic cell lineage in KIRC, LUAD, lung squamous cell carcinoma (LUCS), COAD, BLCA, and READ (Figures 9(a)–9(f)). Moreover, the pathways of cell adhesion molecules, chemokine signalling pathway, autoimmune thyroid disease, type I diabetes mellitus, primary immunodeficiency, and graft versus host diseases were enriched in KIRC and LUAD (Figures 9(a) and 9(b)), and the signalling pathway of asthma, allograft rejection, virtual myocarditis, the internal immune network for IgA production, and *Staphylococcus aureus* infection were enriched in LUCS and READ (Figures 9(c) and 9(f)). Furthermore, GSEA revealed that *C16orf54* expression was significantly related to various immune-related pathways, such as PI3K/Akt/mTOR and WNT/BETA CATENIN signalling pathways in BLCA (Figure 10(a)), IL2/STAT5, inflammatory response, IL6/JAK-STAT3, TNFA/NFKB, and interferon-gamma response signalling pathways in COAD, KIRC, LUAD, LUSC, and READ (Figures 10(b)–10(f)). Interestingly, apoptotic signalling pathways were enriched in various tumours (Figures 10(a)–10(f)). Hence, *C16orf54* is closely related to various immune signalling pathways.

## 4. Discussion

Tumour malignancy is a significant threat to human health and life. In the past, clinical treatments mainly focused on surgery, radiotherapy, and chemotherapy, which included various limitations, such as prominent side effects, drug resistance, easy recurrence, and metastasis. Different from conventional treatment, tumour immunotherapy uses the human immune system to resist the attack and growth of tumour cells. It is currently one of the most promising therapies. Tumour immunotherapy usually includes cellular immunotherapy and immune checkpoint inhibitor therapy [4, 17]. Cellular immunotherapy directly recognises tumour cell surface proteins and induces cancer cell death by stimulating autoimmune cells. However, owing to the heterogeneity of tumour antigens and the failure of tumour-infiltrating T lymphocytes, the effect of cellular immunotherapy in treating solid tumours was unsatisfactory [18]. With the in-depth study of tumour immunotherapy and pathogenesis, immunomodulatory genes have become potential targets of immunotherapy, with immune checkpoint inhibitors widely used as tumour immunotherapy drugs. The immune checkpoint is a significant factor in tumour immune tolerance. The intervention of immune checkpoints can reactivate T cells, thereby inducing tumour cell death [19–21]. CTLA-4 inhibitor and PD-1 inhibitor (PD-1/PD-L1 inhibitor) have strong antitumour activities in different tumours including melanoma, non-small-cell lung cancer and kidney cancer [22, 23]. Although immunotherapy is considered to be the most promising tumour treatment method, its efficacy on different tumour types and patients with the same cancer type varies. As a result, it is of great significance to determine novel tumour immune markers and potential therapeutic targets.

*C16orf54* is a protein-coding gene with heterogeneous expression in different normal and tumour tissues. Expression profile and immunohistochemical results analysis showed that C16orf54 expression in BLCA, BRCA, COAD, LUAD, LUSC, and other tumour tissues decreased significantly, suggesting that *C16orf54* could play a role in the occurrence and development of different tumours. Furthermore, evaluating the association between *C16orf54* expression and OS, DSS, DFI, and PFI showed that *C16orf54* expression was related to the prognosis of many tumours. Among them, low *C16orf54* expression was considered to be a risk factor for SKCM, LUAD, CESC, and other tumours. Liu et al. report that *C16orf54* expression levels were associated with BRCA, LUAD, LGG, and SKCM prognosis, but it remains unclear whether low or high *C16orf54* expression is a prognostic risk factor [24]. The above results show that *C16orf54* is a potential marker of the poor prognosis of various tumours, further suggesting that *C16orf54* participates in the progression of tumours. Analysing the role of *C16orf54* in tumour diagnosis using ROC curve revealed that *C16orf54* could distinguish tumour tissues, such as LAML, TCGT, LUSC, and PAAD, from normal tissues with high accuracy, which suggests C16orf5 as a potential biomarker for tumour diagnosis

The tumour microenvironment (TME) significantly affects the survival, proliferation, immune escape, diffusion, metastasis, and clinical prognosis of tumour [25, 26]. As the main component of the microenvironment, stromal, immune, and estimate scores in the microenvironment change with the interaction between tumour cells and the microenvironment, which has a significant impact on the immune metabolism of tumour cells [27]. The correlation analysis between *C16orf54* expression and TME affirmed that *C16orf54* expression was significantly positively associated with the three microenvironment scores of most tumours and negatively correlated with tumour purity, suggesting that *C16orf54* affects the TME. Interestingly, recent research has revealed that *C16orf54* can affect the morphology of lipid droplets (LDs) that exist in a variety of cells and can be used to generate metabolic energy and cell membrane [28]. This suggests that *C16orf54* may regulate homeostasis of cell energy supply and cell membrane and thereby affect the crosstalk between various cells in the TME of various tumours.

Furthermore, the function of *C16orf54* in the modulation of the tumour immune microenvironment (TIME) was investigated. In addition to tumour cells, TIME also includes nontumour cells, such as fibroblasts, endothelial cells, stromal cells, and immune cells, that interact with tumour cells [29–31]. Immune cells in TIME, such as macrophages, lymphocytes, and NK cells, have a critical impact on tumour immune escape and tumour immunotherapy resistance. Previous studies have reported that the antitumour immune efficacy can be improved by modifying the TIME, such as reconstructing the immune microenvironment by inducing immune cell death in cancer cells, inhibiting glucose metabolism, and repolarising tumour-related macrophages [32]. Therefore, TIME is of great significance in tumour initiation, development, metastasis, and treatment. Immunotherapy shows obvious therapeutic differences among patients, which is largely attributed to the heterogeneity of TIME. Clinical studies have shown that the degree of immune cell infiltration has a critical impact on the prognosis of patients with tumours. Wang et al. report that the tumour-infiltrating IL17+ cells can activate the antitumour response of the TME [33], and M2 macrophages contribute to metastasis and invasiveness of lung and liver tumours [34, 35]. Therefore, immune cell infiltration could be used as an indicator of the prognosis of disease and response to treatment. In the present research, *C16orf54* expression was remarkably positively associated with the infiltration level of different immune cells in most tumour types, especially UVM, UCEC, THYM, and SKCM, suggesting that *C16orf54* can participate in tumour progression and immunotherapy by regulating immune cell infiltration.

The immunomodulatory factor is the medium of communication between infiltrating immune cells and tumour cells. It recruits or expels immune cells into the TME and mediates immune cells to kill or protect tumour cells. The interaction between tumour cells and the immune system is regulated by various immune regulatory factors. Tumour cells inhibit the antitumour immune response and induce immune escape by upregulating immunosuppressive factors or downregulating immune activators [36–38]. Certain positive regulatory factors, such as CD27, CD28, CD30, ICOS, and negative regulatory factors, such as CTLA4, PD-1, BTLA, TIM3, andLAG3, at immune checkpoints affect the recognition and apoptotic ability of the immune system against tumour cells [39–41]. *C16orf54* gene is coexpressed with various immune inhibitors, immune-stimulatory factors, chemicals and chemokine receptors, MHC genes, and other immunomodulatory genes across pan-cancer, suggesting that *C16orf54* can regulate the expression of immunomodulatory genes or it is itself an immunomodulatory gene.

During tumourigenesis and development, the clone type of tumour cells changes constantly due to differences in gene mutation, expression, or methylation [8]. These highly heterogeneous tumour cells give rise to varying responses to various tumour treatments. TMB and MSI are closely related to the prognosis of many tumour types after immunotherapy and can be used as biomarkers to predict immunotherapy efficacy [42]. The tumour remission results and survival benefits after nivolumab treatment were significantly better than those after chemotherapy for patients with high TMB levels [43]. MSI detection is also widely used in the clinical treatment of patients with COAD [44]. This study found that *C16orf54* expression was significantly negatively linked to TMB and MSI in five and ten tumour types, respectively, further suggesting that *C16orf54* could potentially predict patient response to immunotherapy. The relationship between *C16orf54* and tumour stemness was also analysed. Cancer stem cells have the ability to renew themselves and can produce heterogeneous tumour cells. They play an essential function in tumour survival, proliferation, metastasis, and recurrence [45–47]. DNAss is the dryness index derived based on methylation data while RNAss is the stemness indices calculated based on expression data. When the stemness index is closer to 1, the degree of cell differentiation tends to be lower, whereas the stem cells' characteristics become stronger. This study observed that *C16orf54* expression was negatively correlated with DNAss in most tumours, such as THYM, GBM, and LUSC; similarly, it was significantly negatively correlated with RNAss in all tumours except THYM. This indicates low *C16orf54* expression corresponds to strong tumour cell stemness, thus promoting tumour proliferation and metastasis. Furthermore, this correlation can be used as a predictor of the efficacy of immune checkpoint inhibition therapy.

In the end, the molecular mechanisms by which *C16orf54* functions were analysed by KEGG and GSEA. KEGG analysis showed that *C16orf54* could be involved in cytokine receptor interaction, Th1, Th2, and Th17 cell differentiation, T cell receptor signalling pathway, hematopoietic cell lineage, and other signalling pathways. GSEA further revealed that *C16orf54* expression was associated with various immune factor-related pathways, such as PI3K/Akt/mTOR, IL2/STAT5, IL6/JAK-STAT3, TNFA/NFKB, TGF-BETA signalling pathways, E2F targets, and MYC targets-V2. Therefore, enrichment analyses showed that the *C16orf54* gene could participate in the occurrence and progression of tumours via the mechanism of the regulation of immune cell infiltration and immune regulatory factor-related signalling pathways.

## 5. Conclusions

*C16orf54* is a promising new diagnostic, prognostic, immune marker, and therapeutic target. The following work will carry out molecular, cellular, and animal experimentation to verify the conclusions of this study.

## Data Availability

All datasets generated for this study are included in the article/Supplementary Material.

## Supplementary Materials

Supplementary Materials Supplementary Figure 1: the DSS of *C16orf54* by Kaplan–Meier analysis. (A-I) The relationship of *C16orf54* expression and DSS in OV, SKCM, SKCM-M, SARC, CESC, LUAD, LIHC, UVM, and LGG. Supplementary Figure 2: the PFI and DFI of *C16orf54* by Kaplan–Meier analysis. (A-J) The correlation between *C16orf 54* expression and PFI and (K-L) DFI in various tumours.
