# Supplementary material for: Integrated Analysis of C16orf54 as a Potential Prognostic, Diagnostic, and Immune Marker across Pan-Cancer
Source: Dis Markers. 2022 Sep 9;2022:9365046. doi: 10.1155/2022/9365046 (PMC9481382; doi:10.1155/2022/9365046)
Supplement: Supplementary Materials — Supplementary Figure 1: the DSS of C16orf54 by Kaplan–Meier analysis. (A-I) The relationship of C16orf54 expression and DSS in OV, SKCM, SKCM-M, SARC, CESC, LUAD, LIHC, UVM, and LGG. Supplementary Figure 2: the PFI and DFI of C16orf54 by Kaplan–Meier analysis. (A-J) The correlation between C16orf 54 expression and PFI and (K-L) DFI in various tumours. [file 9365046.f1.docx]

**
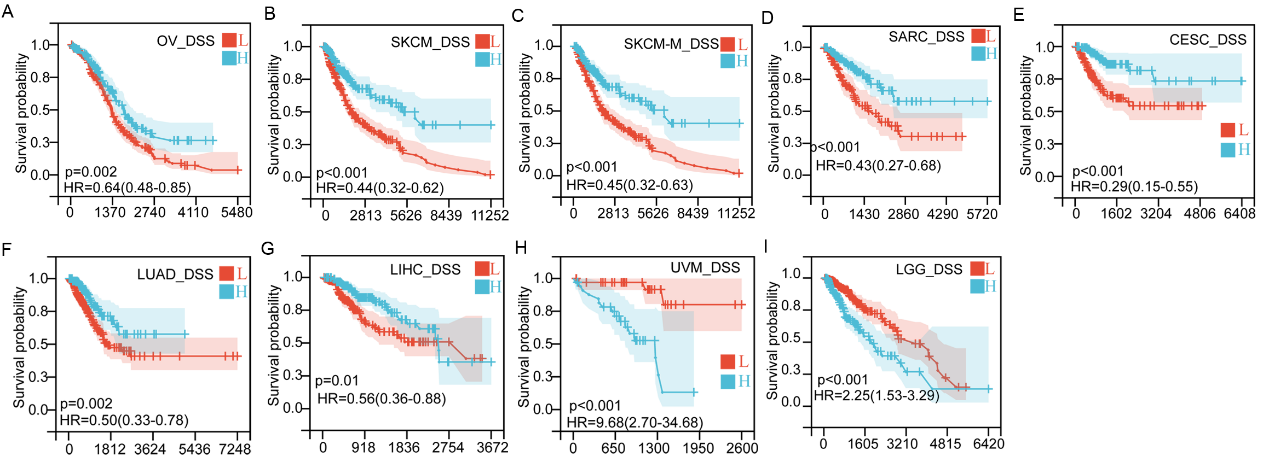
**

**Supplementary Fig.1. The DSS of *C16orf54* by Kaplan–Meier analysis.** (A-I) The relationship of *C16orf54* expression and DSS in OV, SKCM, SKCM-M, SARC, CESC, LUAD, LIHC, UVM and LGG.


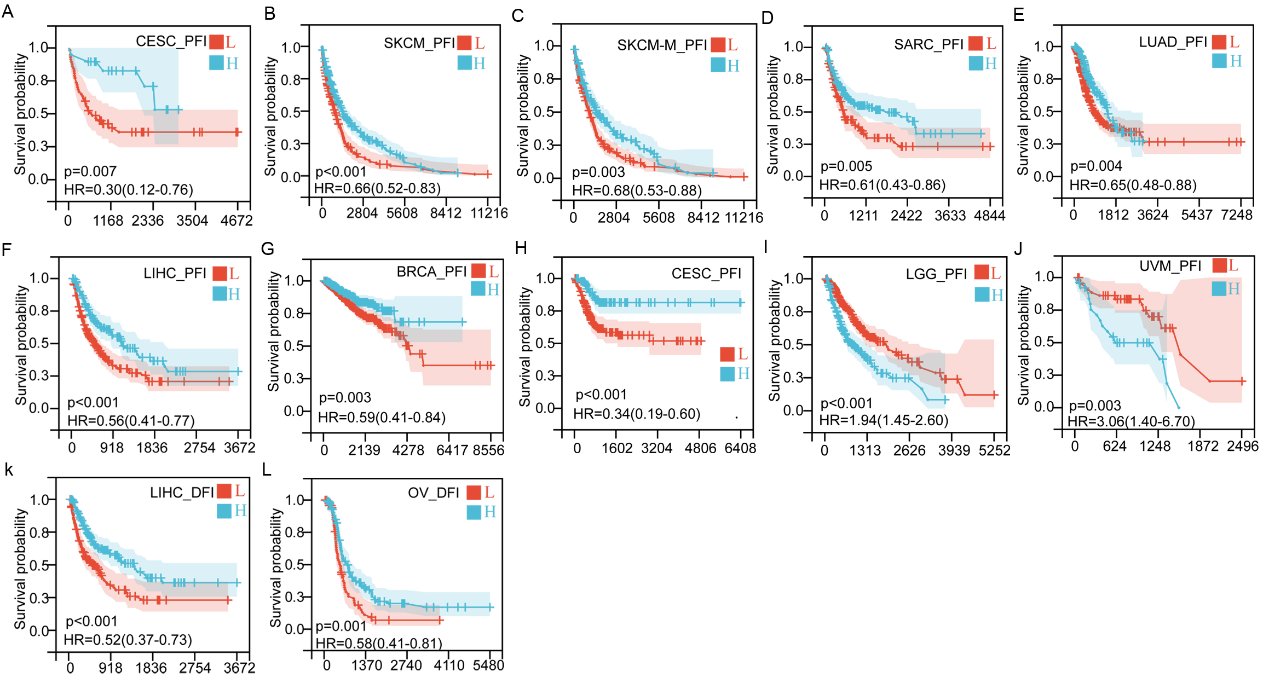


**Supplementary Fig.2. The PFI and DFI of *C16orf54* by Kaplan–Meier analysis.** (A-J) The correlation between *C16orf 54* expression and PFI and (K-L) DFI in various tumours.
